# Treatment of balance with Computerised Dynamic Posturography therapy in chronic hemiplegic patients

**DOI:** 10.4102/sajp.v79i1.1918

**Published:** 2023-09-18

**Authors:** Işıl Doğaner, Zeliha C. Algun

**Affiliations:** 1 Department of Physiotherapy and Rehabilitation, Institute of Health Sciences, Istanbul Medipol University, Istanbul, Turkey; 2 Department of Physiotherapy and Rehabilitation, Yeni Yüzyıl University, Gaziosmanpaşa Hospital, Istanbul, Turkey

**Keywords:** hemiplegia, balance, Computerised Dynamic Posturography, Sensory Organisation Test, chronic hemiplegic falls

## Abstract

**Background:**

As patients with hemiplegia have a high risk of falling, it is important to develop a fall rehabilitation plan and/or apply personalised treatment when necessary.

**Objectives:**

We aimed to evaluate the effects of individualised treatment with Computerised Dynamic Posturography (CDP) on balance in patients with and without a history of chronic hemiplegic falls.

**Method:**

Forty patients with hemiplegia (time post-stroke: 8–18 months) between 40 and 70 years of age in the Istanbul Yeniyüzyıl University, Gaziosmanpaşa Hospital participated in our study. The patients were divided into two groups: Group 1, falling history (*n* = 20) and Group 2, no falling history (*n* = 20). The patients in both groups were included in a traditional rehabilitation programme for 5 weeks, 5 days a week, for 1 h. The group with a history of falls also received individualised CDP treatment for 20 min, 3 days a week, for 5 weeks. Patients were evaluated with a Sensory Organisation Test (SOT) and a Berg Balance Scale (BBS).

**Results:**

In Group 1, a significant improvement was determined in the after-treatment SOT 5 values compared with the before treatment SOT 5 values (*p* = 0.022). Significant improvement was found in BBS (*p* = 0.003) and SOT 6 (*p* = 0.022) values in Group 2. There was no statistically significant difference in improvement between the two groups (*p* ≥ 0.05).

**Conclusion:**

Larger samples and longer duration of individualised CDP therapy studies may be required to improve balance with chronic hemiplegia and a history of falls.

**Clinical Implications:**

In addition to traditional therapy, individualised CDP treatment may be beneficial for patients with a history of post-stroke falls.

## Introduction

Hemiplegia that develops after stroke is a risk factor for falls (Winstein et al. 2016). Movement disorders, decrease in balance, use of psychotropic drugs, inadequacies in self-care, depression and cognitive impairment are among the causes that predispose to falls in patients with hemiplegia (Wei et al. 2019). In patients who develop hemiplegia after stroke, the rate of falling in the first 6 months is 70%. Moreover, fractures involving the hip or pelvic region because of falls are higher in patients with hemiplegia (27%) than in the general population of the elderly (10%) (Wei et al. 2019). Clinical studies reveal that the fear of falling again is between 30% and 80% in patients with hemiplegia (Laren et al. 2018; Schmid et al. 2015; Schmid & Rittman 2007). Balance impairment in people after a stroke is also associated with lower quality-of-life scores (Schmid et al. 2013). Therefore, improving balance is a major goal of stroke rehabilitation.

Various interventions have been used to facilitate balance in people with paralysis such as ankle-foot orthoses, active and passive weight-shifting activities, the use of various feedbacks to improve standing stability, physical exercises and computer-assisted visual feedback training (Aruin et al. 2012; Srivastava et al. 2009; Tsaklis, Grooten & Franzen 2012). Computerised Dynamic Posturography (CDP); (NeuroCom^®^ Balance Manager^®^ Systems, NATUS, balance and mobility, Canada, United States [US]), which was originally used for the evaluation of balance in the laboratory environment and rehabilitation centres, is also now used in the rehabilitation of balance (Galeano et al. 2014; Lendraitiene et al. 2017). Computerised Dynamic Posturography enables quantitative and objective assessment of the sensory and motor components of the balance control system. It is a method in which the balance of an individual is evaluated by using different test positions such as the situations that the patient may encounter in daily life. Initially used in vestibular diseases, CDP is now also used in patients with central nervous system disorders (Bonan et al. 2004). The sensory conflicts used in CDP rehabilitation are like the Sensory Organisation Test (SOT).

The Sensory Organisation Test assesses the patient’s ability to suppress false information to maintain static balance using vestibular, visual and proprioceptive information (Nashner & Peters 1990). Thus, SOT tests the patient under varying sensory conditions. The SOT is frequently used to evaluate the effectiveness of postural control and rehabilitation in many diseases (Badke et al. 2005; Bittar & Barros 2011). The Sensory Organisation Test is currently accepted as the gold standard for the objective assessment of balance related to the use of vestibular, somatosensory and visual inputs in postural control (Pierchala et al. 2019).

In patients with hemiplegia who have a history or risk of falls, a rehabilitation plan for falls is recommended and individualised treatment implemented when necessary (Adeoye et al. 2019; Bakar et al. 2015). In our study, we aimed to evaluate the results of CDP training, which was applied together with traditional rehabilitation in patients with hemiplegia with a history of falling using SOT and the Berg Balance Scale (BBS) as outcome measures.

## Methods

Our prospective study was conducted in the T.C. Istanbul Yeni Yüzyıl University, Gaziosmanpaşa Hospital, Physiotherapy and Rehabilitation Unit, between 12 August 2019 and 12 July 2021, in accordance with the principles of the Declaration of Helsinki. Written and verbal consent from all patients was obtained before they were enrolled. A sample of 36 patients was required for the study to ensure a 5% level of error: 80% power and 10%-unit change. Forty patients were included and were divided into two groups. Group 1 consisted of 20 patients with hemiplegia and a history of falling and Group 2 had 20 patients with hemiplegia and a history of not falling. The aetiology of the patients’ groups were thrombus, embolism and bleeding. Inclusion criteria were determined as being between the ages of 40 years and 70 years, having paralysis for 8–18 months, having a history of falling at least once in the last month for the falling group, absence of visual, auditory, vestibular, neurological and orthopaedic problems that may adversely affect the patients, Brunnstrom lower extremity motor stage *Alt Ekstremite Motor Evresi* (AEME) [Lower Extremity Motor Stage {LEMT}] > 3, Modified Ashworth scale < 3 and having one incidence of stroke only. Those with shoulder pain, those using an orthosis and those with respiratory distress were excluded. No authorisation is required for the use of the CDP device used during the implementation of the test protocol.

### Test protocol

#### Sensory Organisation Test

The Sensory Organisation Test is a valid reliable tool that analyses sensory inputs in six different states (SOT 1 to SOT 6), which can be assessed through the CDP (Lendraitiene et al. 2017; Olchowik et al. 2015). In SOT 1, the eyes remain open, while the platform and the surrounding cabin are fixed. In SOT 2, eyes are closed, while the platform and surrounding cabin are fixed. In SOT 3, eyes remain open and the platform is fixed, while the perimeter cabin oscillates. In SOT 4, eyes remain open, while the platform oscillates, and the perimeter cabin is stationary. In SOT 5, the eyes are closed, while the platform oscillates, and the perimeter cabin is stationary. In SOT 6, eyes remain open, while the platform and the environmental cabin oscillate. The SOT scores range from 0 to 100. A value of zero (0) indicates a risk of falling and a value of 100 indicates perfect stability. The higher the SOT value, the better the stability and balance (Hall & Heusel-Gillig 2010).

#### Berg Balance Scale

The BBS is a valid and reliable scale commonly used in patients with hemiplegia in which functional balance is assessed in the sitting and standing positions. A score between 0 and 4 is applied for each activity performed. The highest score that can be obtained from the BBS is 56. The target score is 45 and scores below 45 indicate balance impairment and fall risk (Winstein et al. 2016).

The assessments were made with the CDP and the SOT and were applied to all participants. The Turkish version of the BBS, whose validity and reliability study was conducted by Şahin et al. in 2008, was used (Sahin et al. 2008).

### Treatment protocols

Traditional rehabilitation included strengthening, active assisted movements and balance exercises using a parallel bar. Balance exercises were performed by stepping forwards, backwards and sideways on the hemiplegic side using a step. Then, patients were trained to walk forwards, backwards and sideways. Traditional rehabilitation was included in the rehabilitation programme for 1 h, 5 days a week for 5 weeks.

Custom training is a training programme incorporated in the software of the CDP device. It is used by physiotherapists following evaluation of the patients and data are entered into the system. It is a rehabilitation programme applied by giving both auditory and visual stimuli to patients with hemiplegia to facilitate the correct transfer of body weight to the side that is affected. The difficulty of the training programme started from 50% and continued by increasing up to 90%. This increase was applied as 10% each week for 5 weeks and 3 days a week. It is 20 min in total and includes four different types of training of 5 min each.

Group 1 and 2 received the given traditional rehabilitation programme for 1 h, 5 days a week for 5 weeks. In addition, Group 1 received individualised treatment with CDP for 20 min, 3 days a week for 5 weeks. The evaluations of patients in Group 1 and Group 2 were performed by the same physiotherapist on the first and the last day of the treatment. The data obtained were recorded for statistical comparison.

### Statistical analysis

The Statistical Package for Social Sciences (Version 22, SPSS Inc, Chicago, Illinois, US) statistical software was used for data analysis. To select the appropriate further statistical analyses, the normality of the distribution of the data groups was determined by the Kolmogorov–Smirnov test. This showed that the data were not normally distributed. Therefore, a Mann–Whitney U test for numeric variables and a Chi-square test for categorical variables were used to compare the data. A Friedman test was used in the evaluation of SOT and BBS, which are repeated measurements within the groups. Descriptive statistics were expressed as mean ± standard value, or categories and percentages of statistical significance were evaluated at the *p* < 0.05 level in all analyses.

## Results

The results of the demographic data of our participants are given in Table 1. There were 11 men and 9 women in Group 1 the falling group, while 7 men and 13 women were in Group 2 the non-falling group. The mean age of the patients in Group 1 was 59.45 ± 8.42 years and in Group 2 was 57.85 ± 11.24 years. There was no difference between the groups in terms of demographic data.

**TABLE 1:** Evaluation of the falling and non-falling patient groups in terms of demographic information.

Variable	Group 1	Group 2	*p*
Age	59.45 ± 8.420	57.85 ± 11.24	0.613*
**Gender**			0.530**
Male			0.530**
*n*	11	7	-
%	55	35	-
Female			
*n*	9	13	-
%	45	65	-
Height	166.05 ± 7.749	167.35 ± 8.35	0.613*
Weight	70.75 ± 9.492	71.80 ± 9.17	0.724*

* , Statistical comparison of measurements between groups with the Mann–Whitney U test.

** , Statistical comparison of measurements between groups with the Chi-square test.

The SOT and BBS results of all participants are shown in Table 2. There was no statistically significant difference between BBS, SOT 1, SOT 2, SOT 3, SOT 4 and SOT 6 scores before and after therapy in Group 1 (*p* > 0.05). However, the post-therapy SOT 5 score (75.46 ± 9.28) improved significantly compared with the pre-therapy SOT 5 score (48.61 ± 7.75) (*p* = 0.022).

**TABLE 2:** Evaluation of Berg Balance Scale and Sensory Organisation Test values of the falling and non-falling patient groups.

Variable	Group 1				Group 2				*p*
	Mean ± s.d.	Min	Max	*p*	Mean ± s.d.	Min	Max	*p*	
**BBS**				0.106***				0.003	0.069*
BT	48.35 ± 7.20	32.00	56.00	-	47.6 ± 8.42	28.00	56.00	-	-
AT	49.55 ± 7.06	38.00	56.00	-	51.05 ± 5.86	38.00	56.00	-	-
**SOT 1**				0.472				0.985	0.694**
BT	92.35 ± 2.90	85.00	96.00	-	91.45 ± 2.98	83.67	95.33	-	-
AT	92.91 ± 2.87	85.00	97.67	-	91.43 ± 2.94	85.67	95.00	-	-
**SOT 2**				0.472				0.968	0.776**
BT	90.67 ± 2.70	83.33	94.00	-	88.68 ± 3.54	79.00	95.00	-	-
AT	91.00 ± 3.65	84.33	98.33	-	88.75 ± 4.33	78.67	96.67	-	-
**SOT 3**				0.207				0.856	0.401**
BT	88.07 ± 4.23	81.33	94.33	-	86.11 ± 4.62	77.00	94.00	-	-
AT	89.89 ± 3.65	82.00	95.67	-	86.60 ± 5.73	71.00	94.33	-	-
**SOT 4**				0.434				0.332	0.892**
BT	79.70 ± 9.13	64.67	93.00	-	75.63 ± 6.92	59.33	85.33	-	-
AT	75.46 ± 9.44	66.33	96.33	-	77.13 ± 11.33	46.67	91.00	-	-
**SOT 5**				0.022				0.218	0.561**
BT	48.61 ± 7.75	52.00	79.67	-	56.43 ± 13.60	17.00	77.33	-	-
AT	75.46 ± 9.28	65.33	98.00	-	60.58 ± 13.43	36.00	90.33	-	-
**SOT 6**				0.983				0.022	0.123**
BT	65.48 ± 10.16	48.00	86.67	-	53.85 ± 11.85	35.33	71.67	-	-
AT	66.70 ± 15.04	41.67	94.67	-	61.17 ± 13.35	34.67	78.00	-	-

Note: *P* significant at ≤ 0.005.

BT, before treatment; AT, after treatment; BBS, Berg Balance Scale; s.d., standard deviation; SOT, Sensory Organisation Test; Min, minimum; Max, maximum.

* , Statistical comparison of measurements between groups with the Mann–Whitney U test.

** , Statistical comparison of measurements between groups with the independent t-test.

*** , Statistical comparison of repeated measurements within groups with the Friedman test, statistical comparison of repeated measurements within groups with the Paired sample t-test.

There was no statistically significant difference between the scores of SOT 1, SOT 2, SOT 3, SOT 4 and SOT 5 before and after therapy in Group 2 (*p* > 0.05). Berg Balance Scale (51.05 ± 5.86) and SOT 6 (61.17 ± 13.35) scores after therapy improved significantly compared with pre-therapy BBS (47.6 ± 8.42) and SOT 6 (53.85 ± 11.85) scores (*p* = 0.003 and *p* = 0.022, respectively). When the SOT and BBS scores were compared between the two groups, no significant difference was observed between the scores.

## Discussion

We examined the effectiveness of traditional therapy and individualised treatment with CDP on balance in patients with hemiplegia with a history of falling. There was a statistically significant difference between the first and second measurements of the SOT 5 scale in the group of patients with a history of falls. There was a statistically significant difference between the first and second measurements of the BBS and SOT 6 scale in the patients who did not have a history of falling.

Balance disorders, which are frequently seen in patients after stroke, cause difficulites in walking and independent daily life and increase the risk of falling (Bourbonnais et al. 2002; Geiger et al. 2001; Laufer et al. 2003). In addition to balance disorders, which can be seen in approximately 87.5% of patients with stroke, these patients may also experience problems such as impaired somatosensory function and coordination, muscle strength and limited range of motion (Oliveira et al. 2011). Restoring balance function in patients with stroke is the most important goal of rehabilitation therapy because serious complications such as traumatic brain injury and bone fracture may develop after a fall (Rubenstein & Josephson 2002). All aspects of postural control (sensory, motor and cognitive) need to be addressed because of the diversity in neurological deficits and differences in compensatory therapies (Fiedorova et al. 2022).

The integrity of the somatosensory system is also very important for motor recovery in patients with hemiplegia. Chen et al. (2018) investigated the therapeutic effects of sensory input training on motor function rehabilitation after stroke. Their study suggests that sensory input has a very important role in the rehabilitation of motor function and that a combined sensory motor training modality is more effective than traditional motor-focused approaches (Chen et al. 2018). One of the important problems affecting balance ability in individuals with hemiparesis after stroke is sensory integration disorder (Bonan et al. 2004). The CDP is used to make an objective and quantitative evaluation of the sensory and motor components that control balance. The CDP targets the motor and sensory systems through its movable support surface and visual environment. Thus, CDP allows testing and training of sensory integration. The reliability of CDP has been described in healthy young and elderly patients, patients with low back pain and transtibial patients (Chien et al. 2007; Dickin 2010; Jayakaran, Johnson & Sullivan 2011). However, there are not enough studies investigating the effect of sensory integration training after stroke using the CDP system (Bayouk, Boucher & Leroux 2006; Smania et al. 2008).

Sensory Organisation Test allows both the objective determination of balance parameters and the identification of movement strategies under certain conditions by somatosensory analysis. Fall estimates with SOT have been studied in people over 65 years of age and in a population with vestibular disorders (Mujdeci, Aksoy & Atas 2012). However, predictive studies have not been adequately performed to predict falls in patients with hemiplegia at high risk of falling after stroke.

In a 2011 study, Oliveira et al. (2011) compared balance in patients with hemiplegia and healthy individuals and found that SOT values of patients with hemiplegia were lower than healthy individuals. They showed that inadequate sensory information significantly affects balance in patients with hemiplegia. They observed that BBS and SOT values of patients with hemiplegia were significantly lower than normal subjects.

It is reported that the history of falling is more common in patients with low balance scores. Maeda et al. (2015) observed that patients in a fallers group were older than patients who were non-fallers and had lower mini-mental state examination score, BBS score and Functional Independence Measure score on admission. Fiedorova et al. (2022) reported that the median age of patients who fell was higher than patients who did not fall, and the median SOT score and median BBS score of patients who fell were lower than the median SOT and median BBS scores of patients who did not fall. Pierchala et al. (2019) documented that rehabilitation provides significant improvements in SOT 4, SOT 6 and composite values in both falling and non-falling groups. Mujdeci et al. (2012) determined that the BBS, SOT 3 and SOT 6 and composite scores of patients with a history of falling were significantly lower than patients without a history of falling. Hakim et al. (2012) observed a general improvement in balance abilities and a reduced risk of falling after a 6-week CDP intervention in a patient with hemiparesis secondary to chronic paralysis.

To the best of our knowledge, the studies evaluating CDP and SOT in the evaluation of balance in patients with post-stroke hemiplegia are limited. In our study, the mean age of the patients in the falling group was numerically higher than the patients in the non-falling group, but this was not a significant difference. In this respect, the age values of the patients in our study are like the findings of other studies. Berg Balance Scale and SOT scores in tests performed before starting therapy were similar in both groups. Other studies have stated that the scores in patients who fall are generally lower than in patients who do not fall. In this respect, the results of our study contradict previous studies. The reason why BBS and SOT scores were similar at the beginning of our study between the falling group and the non-falling group can be explained by the fact that the ages of the patients in both groups were similar and their ages were lower than in other studies. After the therapy applied to both groups, an increase was observed in SOT 5 in patients who had a history of falling and an increase in BBS and SOT 6 in patients who did not fall. Traditional therapy contributed to the results of both groups, as both groups had traditional therapy.

A different result may be achieved by improving the CDP treatment applied to the falling group or by increasing the duration of the treatment applied.

Our study, unlike the results of previous studies, did not show any general improvement after CDP. We believe that this difference is related to the aetiology of the patients, and the duration and frequency of the treatment applied.

The limitations of our study are the single-centre study design and the lack of a group of patients with a history of falling who received traditional therapy only.

## Conclusion

Loss of strength, loss of sensation, visual problems and difficulty in walking may develop in patients with stroke because of sensorimotor dysfunction. Considering these problems, we predict that control over the centre of gravity and purposeful isolated active movements may develop in line with the strategy of somatosensory system work. The authors believe that with a larger number of patients and a longer duration of individualised treatment with CDP the improvement in SOT and BBS parameters measured in patients with hemiplegia with a history of falls may be seen.
